# A Motor Function for the DEAD-Box RNA Helicase, Gemin3, in *Drosophila*

**DOI:** 10.1371/journal.pgen.1000265

**Published:** 2008-11-21

**Authors:** Ruben J. Cauchi, Kay E. Davies, Ji-Long Liu

**Affiliations:** Medical Research Council Functional Genomics Unit, Department of Physiology, Anatomy, and Genetics, University of Oxford, Oxford, United Kingdom; The Jackson Laboratory, United States of America

## Abstract

The survival motor neuron (SMN) protein, the determining factor for spinal muscular atrophy (SMA), is complexed with a group of proteins in human cells. Gemin3 is the only RNA helicase in the SMN complex. Here, we report the identification of *Drosophila melanogaster* Gemin3 and investigate its function in vivo. Like in vertebrates, Gemin3 physically interacts with SMN in *Drosophila*. Loss of function of *gemin3* results in lethality at larval and/or prepupal stages. Before they die, *gemin3* mutant larvae exhibit declined mobility and expanded neuromuscular junctions. Expression of a dominant-negative transgene and knockdown of Gemin3 in mesoderm cause lethality. A less severe Gemin3 disruption in developing muscles leads to flightless adults and flight muscle degeneration. Our findings suggest that *Drosophila* Gemin3 is required for larval development and motor function.

## Author Summary

The childhood disease spinal muscular atrophy (SMA) has a drastic impact on motor neurons and muscles. The cause has been linked to a deficiency in the survival motor neuron (SMN) protein. SMN interacts with various proteins termed Gemins to form the SMN complex, among which Gemin3 is the only one with an RNA unwinding activity. Here, we study the function of *D. melanogaster* Gemin3 in the context of development. The association of Gemin3 with SMN, which had been reported previously in humans, is conserved in flies. Loss of Gemin3 resulted in death at larval stages. Before they die, *gemin3* mutant flies become sluggish and develop large synapses, which are the contacts between motor neurons and muscles. Disruption of Gemin3 in mesodermal tissues, especially muscles, causes development defects, degeneration of flight muscles, and flies that are unable to fly. This study demonstrates that Gemin3 plays a critical role in fruit fly development, especially in motor function, which raises the question of whether disruption of Gemin3 contributes to SMA.

## Introduction

Spinal muscular atrophy (SMA) is an autosomal recessive disorder characterised by degeneration of spinal cord motor neurons, as well as progressive muscular weakness, dysphagia, dyspnoea, and in severe cases, death [1],[2]. The majority of SMA patients harbour deletions or mutations in the *survival motor neuron* (*SMN1*) gene, which encodes an RNA-binding protein, SMN. In mammalian cells, the SMN protein is stably complexed with a group of proteins including Gemin2 [3], Gemin3 [4],[5], Gemin4 [6], Gemin5 [7], Gemin6 [8], Gemin7 [9], and Gemin8 [10]. Biochemical studies in vertebrate systems suggested that the SMN complex plays an essential role in small nuclear ribonucleoprotein (snRNP) assembly. The SMN complex binds directly to small nuclear RNAs (snRNAs) and ensures that a set of seven Sm or Sm-like (Lsm) proteins are assembled onto snRNAs [11].

Gemin3, the only RNA helicase in the SMN complex, contains nine conserved motifs including the Asp-Glu-Ala-Asp motif (or DEAD box in one-letter code). The RNA helicase activity of Gemin3 is ATP-dependent with a 5′ to 3′ direction [12]. RNAi-mediated knockdown studies indicated a role for Gemin3 in the assembly of snRNP complexes as an integral component of the macromolecular SMN complex [13],[14]. Furthermore, a recent study demonstrated that intracellular Gemin3 proteolysis by a poliovirus-encoded proteinase led to reduced Sm core assembly activity in poliovirus-infected cells [14].

In addition to snRNP biogenesis, Gemin3 was also implicated in transcriptional and microRNA regulation. Gemin3 was originally isolated as a cellular factor that associates with the Epstein-Barr virus nuclear proteins EBNA2 and EBNA3C, which play a role in the transcriptional regulation of both latent viral and cellular genes [15]. The non-conserved C-terminal domain of Gemin3 has been shown to interact with and modulate a variety of cellular transcription factors including steroidogenic factor 1 [12],[16], early growth response protein 2 [17], forkhead transcription factor FOXL2 [18], and mitogenic Ets repressor METS [19]. Although the majority of Gemin3 and its associated protein, Gemin4, are found in the SMN complex, a less abundant Gemin3-Gemin4 complex has been isolated from HeLa and neuronal cells. The Gemin3-Gemin4 complex contains Argonaute 2 and numerous microRNAs, co-sedimenting with polyribosomes [20]–[22].

Despite the detailed studies in vertebrate systems and a recent study in *Drosophila* culture cells [23], the function of Gemin3 in *Drosophila* development remains elusive. Here we identify the orthologue of Gemin3 in *Drosophila melanogaster* and demonstrate that *Drosophila* Gemin3, like its vertebrate counterpart, associates with SMN. Loss-of-function *gemin3* mutants are lethal as third instar larvae and/or prepupae. Before they perish, *gemin3* mutants exhibit dramatic loss of mobility and neuromuscular junction (NMJ) defects. Tissue-specific expression of a dominant-negative transgenic construct and RNAi studies suggest that the function of Gemin3 in mesoderm, particularly in muscles, is essential for animal survival. Furthermore, disruption of Gemin3 in muscles causes flight muscle degeneration and loss of flight. Thus our study demonstrates that *Drosophila* Gemin3 plays a critical role in development and motor function.

## Results

### *Drosophila* Orthologue of the Vertebrate Gemin3

**Figure 1:**
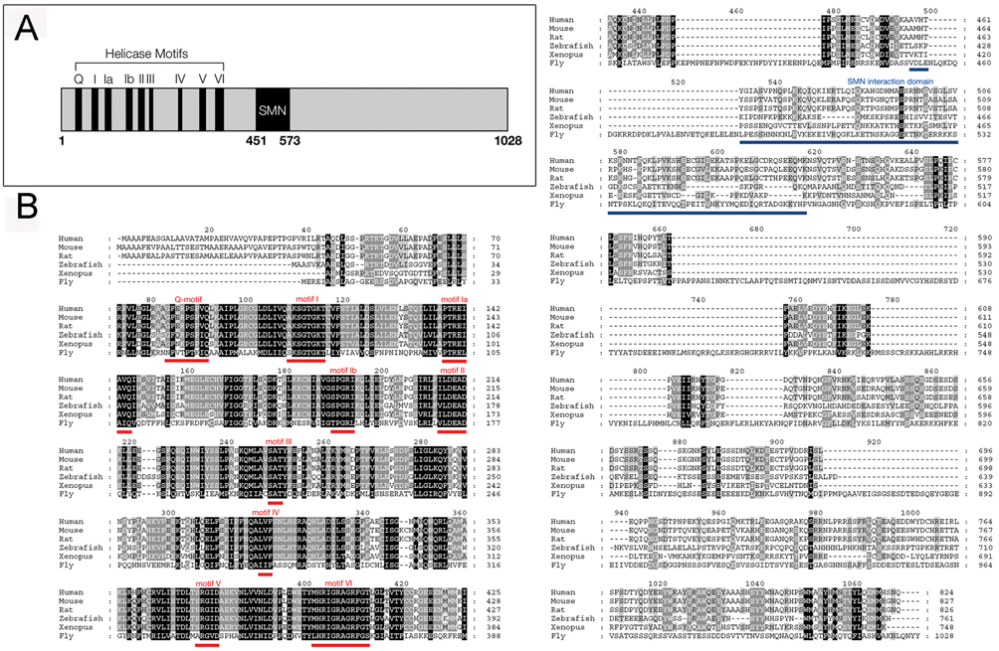
Conservation of *Drosophila* Gemin3. (A) Schematic representation of the *Drosophila* Gemin3 protein sequence showing the helicase motifs, and the SMN interaction domain uncovered in vertebrates. (B) Alignment of the Gemin3 amino acid sequence derived from various species. High identity is observed at the N-termini where all nine DEAD-box helicase motifs (highlighted in red) are conserved. The C-termini, including the region that binds SMN in higher eukaryotes (highlighted in blue), have lower identity. Conserved residues are shown in light grey (weakly conserved) to black (highly conserved).

We carried BLAST searches of the *Drosophila melanogaster* genome using human and mouse Gemin3 sequences, and found that the DEAD/DEAH RNA helicase 1 (Dhh1) or CG6539 is the putative *Drosophila* Gemin3 orthologue. This gene, renamed for the present studies as *gemin3*, is located on the third chromosome in region 67E3, and is composed of 2 exons separated by a short intron. The *Drosophila melanogaster* Gemin3 protein is composed of 1028 amino acids and shows 33% identity and 55% similarity (BLASTP) to the respective human orthologue (Figure 1A, B). This level of conservation is quite similar to that observed between the *Drosophila* and human SMN, which have an overall identity and similarity of 31% and 49%, respectively. The N-termini of Gemin3, in which all nine DEAD-box helicase motifs reside, are more conserved than the C-termini. A region in the middle (451–573aa) of *Drosophila melanogaster* Gemin3 corresponds to the SMN-binding domain identified in higher eukaryotes [5].

**Figure 2:**
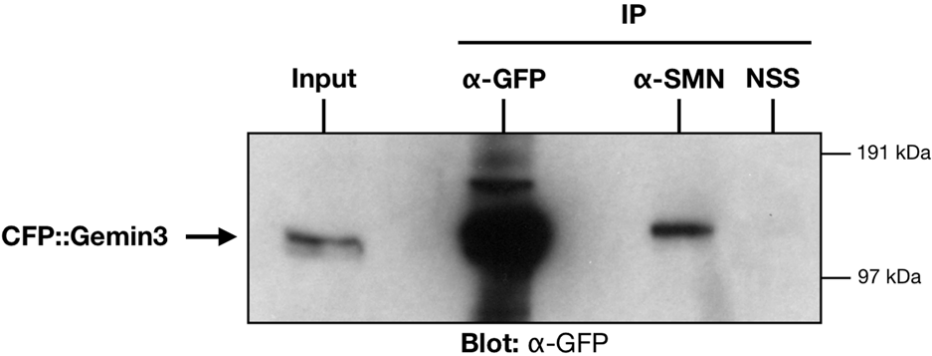
In vivo association of *Drosophila* Gemin3 and SMN. Western blot probed for CFP::Gemin3 fusion protein (∼143 kDa) using mouse anti-GFP antibody after immunoprecipitation (IP) with rabbit anti-GFP antibody (positive control), rabbit anti-SMN (test) or rabbit non-specific serum (NSS; negative control). Protein extracts were derived from larval muscles ubiquitously expressing CFP::Gemin3. CFP::Gemin3 co-purifies with SMN, but is absent in the negative control lane. Input was one-tenth volume of the whole tissue lysate.

Aiming to test whether the physical interaction between SMN and Gemin3 reported in higher eukaryotes [24] is conserved in *Drosophila*, a co-immunoprecipitation approach was pursued. We have generated a transgenic line expressing CFP::Gemin3. The CFP::Gemin3 gene is functional as it can rescue *gemin3* mutants, which we describe later. In extracts derived from CFP::Gemin3 transgenic larvae, anti-SMN antibodies co-immunoprecipitate CFP::Gemin3 (Figure 2).

### *gemin3* Is an Essential Gene

**Figure 3:**
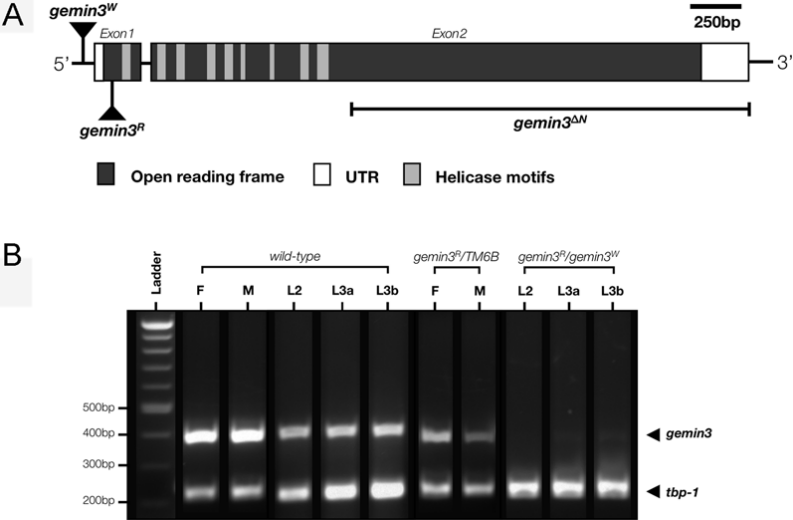
Alleles of *gemin3*. (A) Schematic showing the location of the *gemin3* alleles and the span of the *gemin3^ΔN^* transgene. The *gemin3^W^* allele is a transposon insert within the *gemin3* gene promoter, specifically at 92 nucleotides upstream of the transcription start site. The transposon insert generating the *gemin3^R^* allele is located 108 and 35 nucleotides downstream of the transcription and translation start site, respectively, thereby generating several premature stop codons. The *gemin3^ΔN^* transgene lacks 424 amino acid residues from the N-terminal of Gemin3 and is thus devoid of the helicase core. (B) RT-PCR analysis of *gemin3* expression in wild-type, *gemin3^R^/gemin3^W^* transheterozygotes and *gemin3^R^* heterozygotes. Compared to wild-type, *gemin3* mRNA was detected in low amounts in *gemin3^R^* heterozygotic adults. Importantly, *gemin3* mRNA expression levels were drastically reduced in *gemin3* heteroallelic mutant larvae. The housekeeping *Tat-binding protein-1* (*Tbp-1*) RT-PCR product served as an internal control. Developmental stages at 25°C: F = adult females, M = adult males, L2 = 48 hrs after egg laying (AEL) 2^nd^ instar larvae, L3a = 72 hrs AEL 3^rd^ instar larvae, and L3b = 96 hrs AEL 3^rd^ instar larvae.

Two recessive lethal *gemin3* alleles were identified: *PBac*{RB}e03688 (*gemin3^W^*) and *P*{PZ}Dhh1^rL562^ (*gemin3^R^*). We used PCR to confirm that the transposon insertion site of the *gemin3^W^* allele is located at 92 nt upstream of the transcription start site (Figure 3A; Figure S1). Part of the 5′ and 3′ *piggyBac* ends in the *gemin3^W^* allele were found to have been lost during the insertion. In the *gemin3^R^* allele, the *P* element inserted at 108 nt downstream of the transcription start site (Figure 3A; Figure S1). Since the *P*{PZ}-element insert sequence generates several premature stop codons, *gemin3^R^* is hypothesised to be an amorph.

Several studies were pursued to demonstrate that the recessive lethality of both transposon insertions is specific to *gemin3* disruption, thereby confirming that *gemin3* is an essential gene. First, complementation crosses revealed that both *gemin3* alleles retain their recessive lethality in trans to each other and to a chromosomal deficiency that completely eliminates the *gemin3* gene (Df[3L]ED4457). Second, a re-mobilisation screen of the *P*-element in the *gemin3^R^* allele, which is the only transposon that could be excised, recovered homozygous viable precise excision alleles or revertants. Third, both low ubiquitous *gemin3* and *CFP::gemin3* transgenic expression driven by *1032*-GAL4 [25] rescued the lethality of *gemin3^R^* homozygotes and *gemin3^R^/gemin3^W^* transheterozygotes. However, neither of the above *gemin3* transgenes can rescue the lethality of homozygous *gemin3^W^*, suggesting that a non-specific mutation may be causing the lethality associated with the *gemin3^W^* allele. Since the lethality observed in *gemin3* heteroallelic mutants was specific to the loss of *gemin3*, further analysis concentrated on this genotype.

Expression of the *CFP::gemin3* transgene under the control of tissue-specific drivers such as *G7*-GAL4 (muscle), *elav*-GAL4 (neuron), or the combination of both could not rescue the lethality of *gemin3^R^* homozygotes and *gemin3^R^/gemin3^W^* transheterozygotes, suggesting that animal survival also depends on the basal level of Gemin3 in tissues not covered by the expression of *G7*-GAL4 or *elav*-GAL4 drivers. Homozygous *gemin3^R^* mutants survive to the third instar larval stage, while the transheterozygotic *gemin3^R^/gemin3^W^* animals survive to the prepupal stage after both genotypes experience a prolonged wandering third instar larval stage. The expression of *gemin3* at different developmental stages was compared by two-step RT-PCR. Essentially *gemin3* mRNA was expressed at all developmental stages (Figure 3B). Supporting the amorphic allele hypothesis, we observed that expression of *gemin3* mRNA was dramatically reduced in transheterozygous animals throughout their entire larval life, whereas the housekeeping control *Tat-binding protein-1* (*Tbp-1*) transcripts remained detectable (Figure 3B). Heterozygous *gemin3^R^* adults have approximately half of the *gemin3* mRNA transcript as that in wild-type animals (Figure 3B).

### Motor Defects in *gemin3* Mutant Larvae

**Figure 4:**
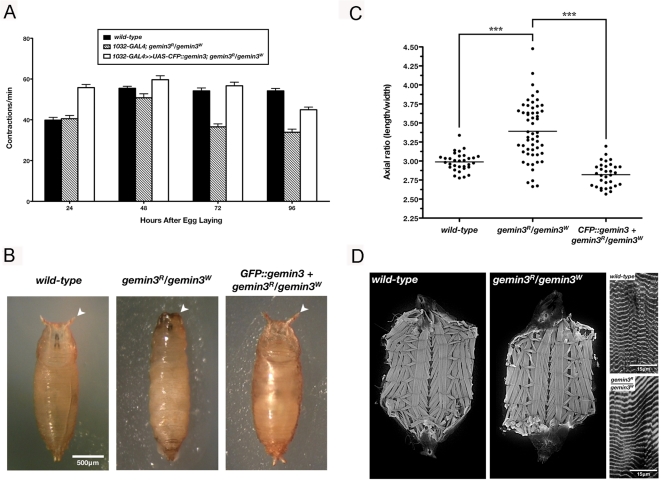
Loss of *gemin3* disrupts larval locomotion and puparium formation. (A) Body wall contraction rate of wild-type (*y w*), *gemin3* heteroallelic mutants (*1032-GAL4*; *gemin3^R^/gemin3^W^*), and rescued *gemin3* mutants (*1032-GAL4≫UAS-CFP::gemin3*; *gemin3^R^/gemin3^W^*) throughout their larval development. Compared to controls, *gemin3* transheterozygotes exhibit a significant reduction in body wall contraction at the third instar larval stage. At 25°C, 24 hrs AEL = 1^st^ instar larvae, 48 hrs AEL = 2^nd^ instar larvae, and 72–96 hrs AEL = 3^rd^ instar larvae. Individual bars represent the mean body wall contraction rate ± 1.0 S.E.M. (*n* = 15–51). (B) Puparia of wild-type, *gemin3* heteroallelic mutants and rescued mutants. Mutants form slender and longer puparia compared to wild-type, a defect that was corrected upon expression of *gemin3* driven by the constitutive driver, *1032-GAL4*. Arrowheads show failure in spiracle eversion. (C) Graph displaying the axial ratios for puparia of the indicated genotype. Compared to wild-type and rescued mutants, axial ratios of mutant puparia were significantly larger. The mean is marked by a horizontal line running through the data points (***p<0.0001; *n*>31). (D) Wild-type and mutant third instar larval muscle fillets labelled using Alexa Fluor-488-conjugated phalloidin show no apparent disruption in the gross structure of body wall musculature (left panels) and sarcomere organisation (right panels).

Although showing no dramatic mobility changes throughout the first and second larval stages, the *gemin3^R^/gemin3^W^* transheterozygotes exhibit a significantly decreased contraction rate at the third instar larval stage (Figure 4A and Video S1). The puparium formed by *gemin3* heteroallelic mutants exhibited failed eversion of the spiracles and a large axial ratio (Figure 4B, C), the latter of which is most probably the result of a failure in body wall muscle contraction. Ubiquitous expression of the *CFP::gemin3* transgene within this mutant background rescues the defects in mobility, spiracle eversion and abnormal axial ratio, confirming that the *CFP::gemin3* transgene is functional and the above phenotypes exhibited by *gemin3^R^/gemin3^W^* transheterozygotes are specifically due to the disruption of Gemin3 function (Figure 4A–C). Mobility failure is probably not secondary to compromised muscle structure since *gemin3* mutant larval fillets have an ordered pattern of muscle fibres without obvious muscle losses. In addition, there are no gross defects in the sarcomeric organisation in the *gemin3* mutants (Figure 4D).

**Figure 5:**
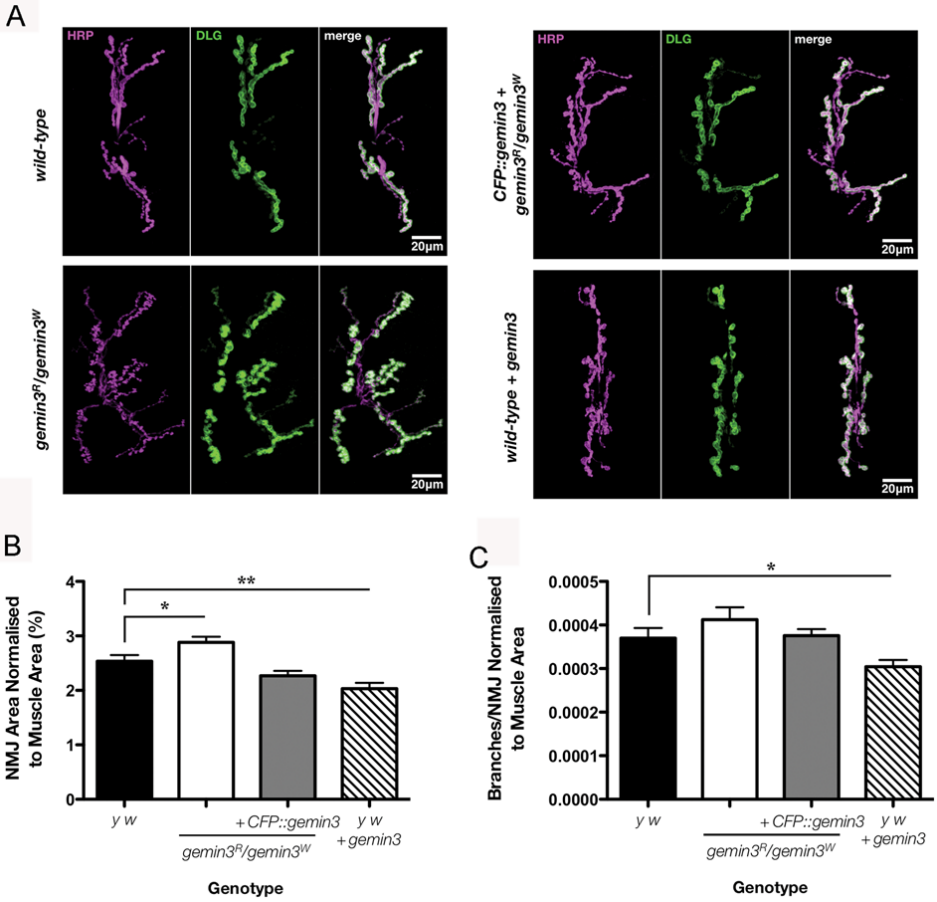
Synaptic growth is influenced by Gemin3 levels. (A) Projections of representative confocal images of NMJs innervating ventral longitudinal muscles 6 and 7 co-stained with anti-HRP (magenta) and anti-DLG (green) in late third instar larvae of wild-type (*y w*), *gemin3* transheterozygotes (*gemin3^R^/gemin3^W^*), *gemin3* transheterozygotes with a ubiquitous expression of *gemin3* (*1032-GAL4≫UAS-CFP::gemin3*; *gemin3^R^/gemin3^W^*), and wild-type with an additional copy of *gemin3* expressed ubiquitously (*1032-GAL4≫UAS-gemin3*). Compared to wild-type and rescued *gemin3* mutants, visual inspection often reveals overgrown NMJs on *gemin3* loss-of-function and undergrown NMJs on *gemin3* gain-of-function. (B, C) Quantification of the area and the number of branches characteristic of NMJs normalised to the area of both muscle 6 and 7 in wild-type (*n* = 25), mutant (*n* = 21), rescued mutant (*n* = 42) and wild-type animals with an additional copy of *gemin3* (*n* = 23). Individual bars represent the mean and error bars represent ± 1.0 S.E.M. (*p<0.05; **p<0.005).

The obvious larval contraction defects of the *gemin3* transheterozygotic mutants directed the research focus on the larval neuromuscular junction (NMJ). The present studies focus on the highly characterised type I NMJ innervating ventral longitudinal muscles 6 and 7, and aim at unveiling the presence of any morphological abnormalities in a *gemin3* mutant background. To this end, larval muscle fillets were dissected and double-labelled with anti-HRP antibodies, which allow visualisation of the neuronal membrane, and an antibody against Discs-large (Dlg), a primarily postsynaptic scaffold protein localised to the subsynaptic reticulum that surrounds each bouton. Although no obvious motor neuron denervation was detected, *gemin3* heteroallelic mutants exhibit an appreciative synaptic overgrowth before pupariation (Figure 5A) and a significantly increased synaptic area even when normalised to muscle size (Figure 5B). Expression of a *gemin3* transgene in a mutant or wild-type background resulted in an increase in both NMJ and muscle area (data not shown). When normalized to muscle area, the NMJ area and branches in rescued *gemin3* mutants restore to the wild-type range, whereas normalized NMJ area and branch numbers within single NMJs are significantly decreased when *gemin3* was overexpressed (Figure 5B, C).

### Mesodermal *gemin3* Is Critical for Animal Survival

**Figure 6:**
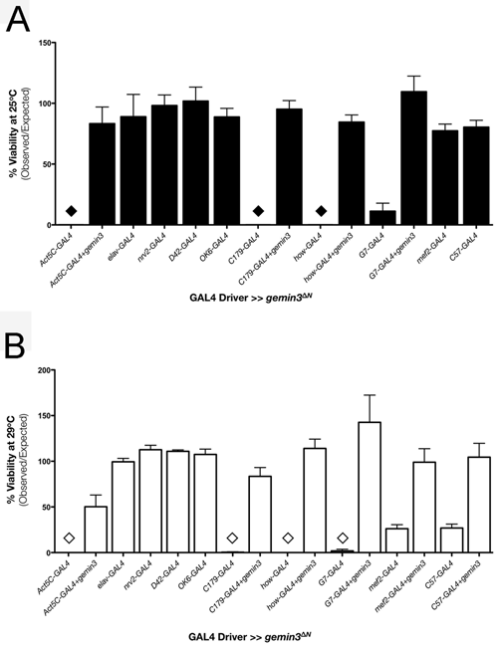
Gemin3 disruption in mesoderm and larval muscles has a drastic impact on adult viability. Bar charts showing adult fly viability assayed at 25°C (A) and 29°C (B). Fly viability is unaffected when the *gemin3^ΔN^* transgene is driven in post-mitotic neuronal tissues via *elav*-GAL4, *nrv2*-GAL4, *D42*-GAL4 and *OK6*-GAL4. Lethality is however obvious when Gemin3 is disrupted in all tissues via *Act5C*-GAL4 or in mesoderm and larval muscles through expression by *how*-GAL4 and *C179*-GAL4. A significant reduction in viability was also observed when the *gemin3^ΔN^* was driven in the muscles via *G7*-GAL4 and at 29°C via *C57*-GAL4. When highly expressed at 29°C, *mef2*-GAL4, which expresses in mesoderm and larval muscles, also has a significant effect on viability. Driver-associated lethality was rescued on co-expression of a full-length *gemin3* transgene. Individual bars represent the mean viability ± 1.0 S.E.M. of 4 independent experiments. The ♦ and ⋄ symbols indicate lethality.

A truncated *gemin3* transgene (*gemin3^ΔN^*), which lacks 424 amino acid residues from the N-terminus of *Drosophila melanogaster* Gemin3 and hence lacks the helicase core (Figure 3A), causes lethality on ubiquitous expression. Whilst highlighting the importance of the helicase domain to the function of Gemin3, the N-terminal truncated Gemin3 isoform is hypothesized to be a dominant-negative mutant. We used various drivers to investigate the effect on animal survival when *gemin3^ΔN^* is expressed in various temporal and spatial expression patterns (Table 1). No dramatic effect is observed when *gemin3^ΔN^* is expressed at 25°C under the control of *elav*-GAL4, *nrv2*-GAL4, *D42*-GAL4, *OK6*-GAL4, *mef2*-GAL4, or *C57*-GAL4 drivers (Figure 6A). However, expression of *gemin3^ΔN^* at 25°C by *Act5C*-GAL4, *how*-GAL4 or *C179*-GAL4 driver results in lethality, and that by the *G7*-GAL4 driver leads to a significant decrease in viability (Figure 6A). When the temperature shifted to 29°C to allow for maximal GAL4 activity, expression of *gemin3^ΔN^* by *Act5C*-GAL4, *C179*-GAL4, *how*-GAL4, or *G7*-GAL4 driver causes lethality, while that by *mef2*-GAL4 and *C57*-GAL4 drivers results in decreased viability (Figure 6B). Co-expression of an extra full-length *gemin3* transgene but not a control gene such as GFP with the *gemin3^ΔN^* transgene significantly alleviates the driver-associated lethality (Figure 6 and data not shown). These experiments indicate that the lethality or low viability associated with the expression of *gemin3^ΔN^* in the mesoderm and larval muscles is specifically due to the disruption of Gemin3 function.

**Table 1:** Documented spatial and temporal expression patterns of GAL4 drivers used in the present study.

Driver name	Expression pattern	References
*Act5C*-GAL4	Ubiquitous expression with an early onset	[38]
*elav*-GAL4	Expression in all postmitotic neurons with an early onset	[39],[40]
*nrv2*-GAL4	Nervous system-specific expression from embryo to the adult stage	[41]
*d42*-GAL4	Larval and adult stage motor neuron-specific expression	[42],[43]
*OK6*-GAL4	Expression in all motor neurons, salivary glands, wing discs, and a subset of tracheal branches commencing in the first instar larval stage and persisting until pupation	[44]
*C179*-GAL4	Expression in mesoderm and larval muscles	[45]
*how*-GAL4	Expression in mesoderm and larval muscles	[46],[47]
*mef2*-GAL4	Expressed in mesoderm, embryonic stage 12 myoblasts and larval muscles	[48],[49]
*C57*-GAL4	Expression observed in all larval muscles from mid-first to third instar larval stage, two sensory cell bodies in the body wall and in other mesodermal tissues including the gut	[50]
*G7*-GAL4	Expression in all muscles beginning from the second instar larval stage	[51]–[53]

**Figure 7:**
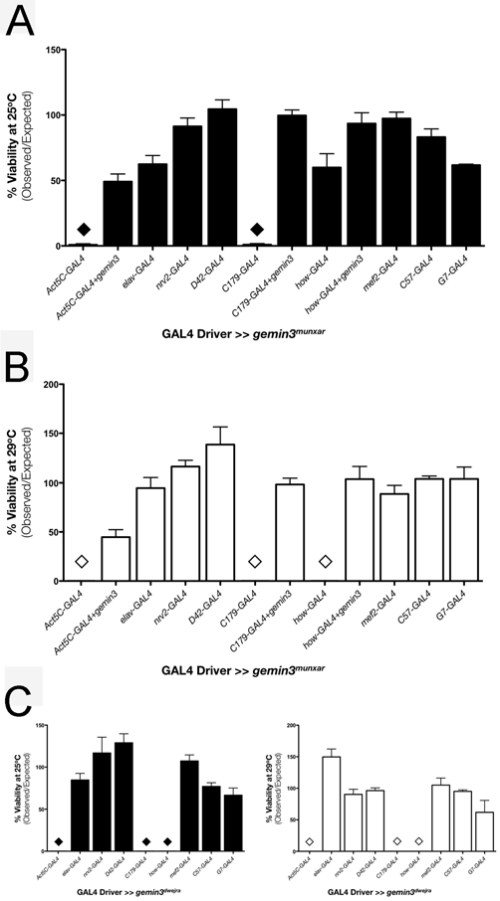
Gemin3 knockdown in mesoderm and larval muscles affects adult viability. (A, B) Knockdown of Gemin3 through expression of the *gemin3^munxar^* RNAi construct in all tissues via *Act5C*-GAL4 or in mesoderm and larval muscles via *C179*-GAL4 and *how*-GAL4 at 29°C (B) leads to lethality. No effect on adult viability was observed when Gemin3 was knockdown in post-mitotic neuronal tissues via *elav*-GAL4, *nrv2*-GAL4, *D42*-GAL4 and *OK6*-GAL4. Driver-associated lethality was rescued on co-expression of a full-length *gemin3* transgene. (C) Similarly, knockdown of Gemin3 via an independent *gemin3* RNAi construct (*gemin3^dwejra^*) induces lethality when expression is driven ubiquitously (*ActC*-GAL4) or in a strong mesodermal and larval muscle pattern (*C179*-GAL4 and *how*-GAL4). Both gemin3 RNAi transgenes targeted the 5′ end sequence of the *gemin3* mRNA transcript. Individual bars represent the mean viability ± 1.0 S.E.M. of 4 independent replicates. The ♦ and ⋄ symbols indicate lethality.

To confirm the driver-specific lethality pattern induced by the *gemin3^ΔN^* transgene, several *gemin3* RNAi transgenic flies were isolated and tested to establish whether lethality can be induced when *gemin3* knockdown occurs ubiquitously throughout the entire organism. Two RNAi transgenes, *gemin3^dwejra^* and *gemin3^munxar^*, fit this criterion. Reducing *gemin3* gene activity using *elav*-GAL4, *nrv2*-GAL4, or *D42*-GAL4 has no effect on fly viability (Figure 7). In contrast, Gemin3 knockdown at both 25°C and 29°C via *C179*-GAL4 resulted in lethality. The *how*-GAL4 driver gave a similar effect when the *gemin3^dwejra^* and *gemin3^munxar^* RNAi transgene was expressed at both temperatures or at a temperature of 29°C, respectively (Figure 7). The lethality induced by *gemin3^munxar^* could be rescued by co-expressing a functional *gemin3* transgene, thus excluding the possibility that lethality is the result of ‘off-target’ effects (Figure 7A, B).

### Driver-Specific Gemin3 Disruption Results in Pupal Developmental Defects or Loss of Flight

**Figure 8:**
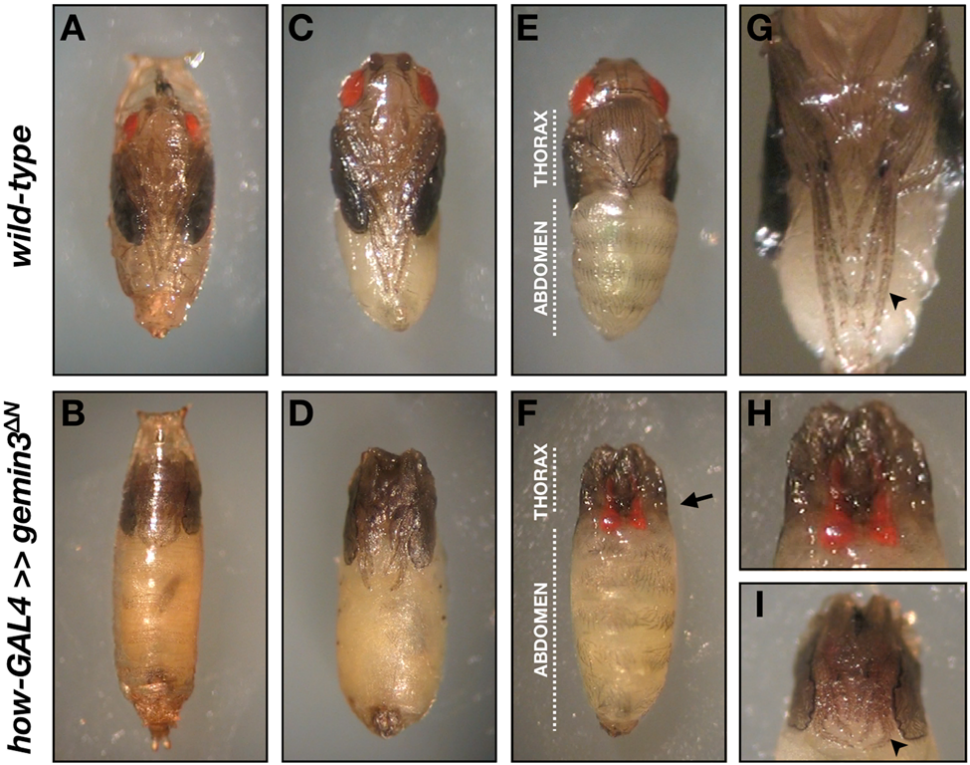
Pupal developmental defects associated with Gemin3 disruption in mesoderm and larval muscles. Compared to wild-type, flies expressing *gemin3^ΔN^* in mesoderm and larval somatic muscles display several aberrations at metamorphosis. (A–D) Ventral view showing that *how*-GAL4≫*gemin3^ΔN^* flies are ‘headless’ as a result of failed head eversion and exhibit elongated pupae as well as shorten wings. (E–F) Dorsal view showing that in *how*-GAL4≫*gemin3^ΔN^* flies the abdomen remains elongated as in the larval stage, and red-pigmented eye discs are often clearly visible within the thorax, denoted by an arrow. The abdomen shows normal segmentation as discerned by the complete bristle pattern on the abdominal tergites. (G–I) Dorsal view showing that compared to controls, in *how*-GAL4≫*gemin3^ΔN^* flies, leg discs have everted but the appendages do not appear fully elongated compared to those of controls (arrowhead). In (C–I), the puparium was removed whilst the pupal cuticle was left intact. Pupae were photographed at the same magnification at approximately 3 days following puparium formation.

Knockdown of *gemin3* in the mesoderm and larval somatic musculature results in lethality at the late pupal stage, that is, pharate adults enclosed in pupae fail to eclose. Animals expressing *gemin3^ΔN^* under the control of the *how*-GAL4 driver often lead to pupariation and puparia have increased axial ratios, similar to the defects exhibited by the *gemin3^R^/gemin3^W^* transheterozygotes. In addition, *how*-GAL4≫*gemin3^ΔN^* pupae exhibited several morphological abnormalities, including head eversion defects, short legs, and short wings, although segmentation of the abdomen and mature eye pigments appear normal (Figure 8).

**Figure 9:**
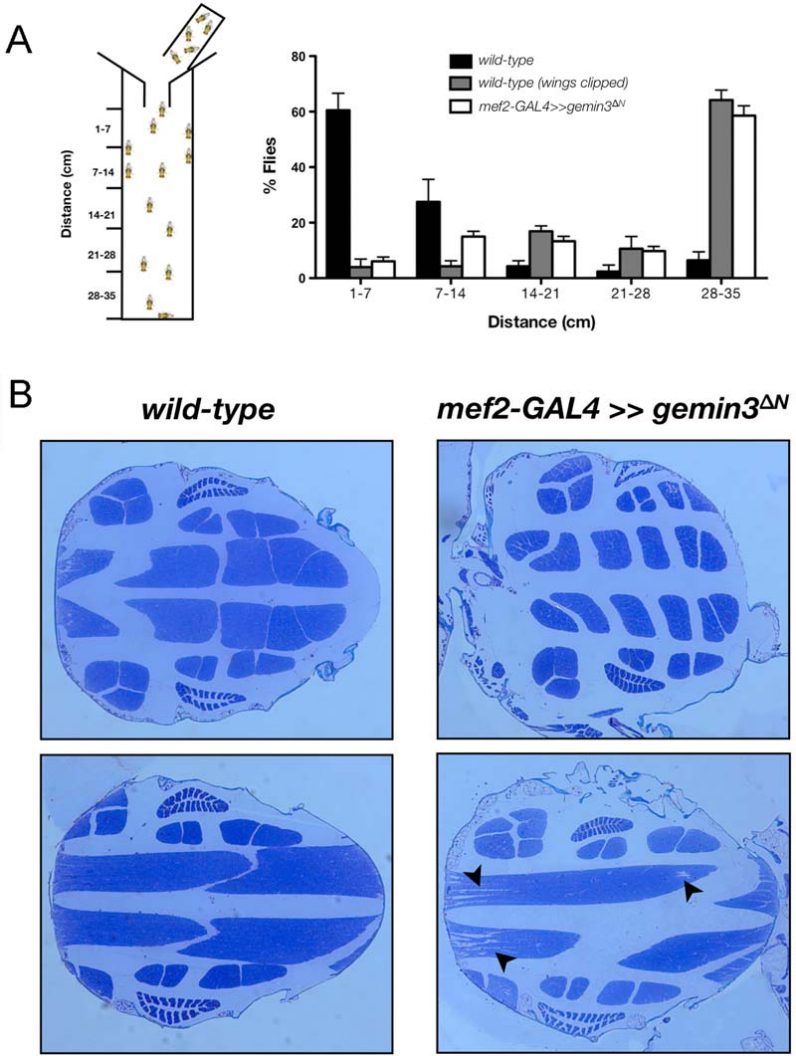
Disruption of mesodermal and muscle Gemin3 leads to loss of flight and flight muscle degeneration. (A) Left shows a schematic diagram of the apparatus for the flight assay. Flies are introduced at the top of the cylinder through a funnel. The height flies stick in the mineral oil coating the inside wall of the cylinder is an indication of their flight ability. Right shows a bar chart quantifying the flight performance of the indicated genotypes. Wild-type flies fly well and thus concentrate at the top of the cylinder. Flies with a *mef2*-GAL4 driven expression of *gemin3^ΔN^* and wild-type flies with clipped wings are skewed towards the bottom of the cylinder, hence illustrating their perturbed flight ability. Five independent assays were performed per genotype with 20 flies in each assay. Flies were approximately one week old. (B) Light microscopic images of sectioned resin-embedded thoraces from 3-week-old adult flies of the indicated genotype. Sections were stained with toluidine blue to visualise the tissue morphology and were taken at the same magnification. Anterior is left. Compared to wild-type controls, *mef2*-GAL4 driven expression of *gemin3^ΔN^* results in flight muscles, which are shrunken, and exhibit several breakages and large tears (arrowheads).

While they can walk and jump normally, eclosed flies with an *mef2*-GAL4-driven *gemin3^ΔN^* expression have a reduced ability to fly. In a flight assay, those flies show defective flight ability, similar to wild-type flies with clipped wings, which are flightless (Figure 9A and Video S2). The indirect flight muscles (IFMs) in *mef2*-GAL4≫*gemin3^ΔN^* flies are shrunken, resulting in increased spacing, and breakages are obvious between the muscle fibers. Frequently, large tears within the indirect flight muscles are observed in *mef2*-GAL4≫*gemin3^ΔN^* flies but not in wild-type flies (Figure 9B).

## Discussion

We have shown that *CG6539*, the *Drosophila* orthologue of vertebrate Gemin3, plays critical roles in larval and pupal development, especially in motor function.

### *CG6539* Is the *Drosophila* Orthologue of Vertebrate *gemin3*

Gemin3 or DP103 was first identified in mammalian culture cells through biochemical approaches [5],[15]. The Gemin3 protein has three critical features. First, the N-terminus of Gemin3 contains multiple helicase motifs including a DEAD-box. Second, Gemin3 interacts with SMN in vitro and in vivo [24]. Third, the Gemin3 and SMN proteins have a similar subcellular localization pattern [5],[26].

In *Drosophila* there are 29 DEAD-box RNA helicases [27]. Using human and mouse Gemin3 to BLAST the *Drosophila melanogaster* genome, CG6539, previously identified as DEAD/DEAH RNA helicase 1 (Dhh1), is the top hit. In the N-terminus, CG6539 contains 9 conserved RNA helicase motifs including a DEAD-box. A segment in the middle of CG6539, which corresponds to the SMN-binding domain in human Gemin3, is less conserved. Moreover, co-immunoprecipitation experiments using *Drosophila* larval muscle extracts show that Gemin3 binds to SMN in vivo. We have also carried localization assays, which demonstrate that Gemin3 co-localizes with SMN in the cytoplasm and nucleus [28] (RJC, KED, and JLL, unpublished data). Taken together, we feel confident that we have identified the *Drosophila* orthologue of vertebrate Gemin3.

Recently, an independent study by Fischer and colleagues also identified CG6539 as *Drosophila* Gemin3 through bioinformatic and biochemical approaches using *Drosophila* culture cells [23]. Both their study in *Drosophila* culture cells and this study in *Drosophila* tissues have shown that Gemin3 interacts with SMN, suggesting that Gemin3 is a *bona fide* component of the SMN complex in fruit flies, similar to that in vertebrate systems.

### *Gemin3* Mutants in Fly and Mouse

In this study, we have multiple lines of evidence demonstrating that *Drosophila* Gemin3 is essential for animal development and survival. Firstly, homozygous loss of *gemin3* through a specific transposon insert (*gemin3^R^*) or a transheterozygous combination of two transposon inserts which do not complement each other (*gemin3^R^/gemin3^W^*) results in lethality at the larval and/or prepupal stage. Secondly, a functional *gemin3* transgene specifically rescues the lethality and developmental defects caused by loss of *gemin3*. Thirdly, expression of a dominant-negative allele of *gemin3* (*gemin3^ΔN^*) or Gemin3 knockdown by RNAi ubiquitously or even in a tissue-specific pattern results in lethality or reduced viability.

Gemin3-null mutants have recently been described in the mouse [29]. Heterozygous *gemin3* mutant mice are healthy and fertile, with minor defects in the female reproductive system, whereas homozygous *gemin3* knockout in mice leads to death at the 2-cell embryonic stage [29]. Thus, the lethality caused by loss of Gemin3 in *Drosophila* is consistent with the findings in Gemin3-null mice. However, while Gemin3-null mice died at an early embryonic stage, *gemin3* mutant flies exhibit delayed lethality, which probably results from maternal contribution of the *gemin3* transcript. In a separate study in female ovaries, we observed severe defects in nurse cells and oocytes when *gemin3* is disrupted in germline cells (RJC, KED, and JLL, unpublished data).

### Motor Function of Gemin3: Pre- or Post-Synaptic?

The earliest clues pointing towards a motor function were a progressive loss of mobility and consequent long and thin puparia when Gemin3 function is lost. Similar phenotypes have previously been observed in mutants with disrupted Mlp84B, a muscle sarcomeric protein [30], or Tiggrin, an extracellular matrix ligand for the position-specific 2 integrins [31]. We also observe that *gemin3* mutants have an overgrown NMJ though these could be a secondary response to the progressive loss of muscle power. The size ratio of NMJs to muscles is reduced when *gemin3* is overexpressed raising the possibility that Gemin3 might also play a role in synaptic growth.

The requirement of Gemin3 in mesoderm and larval muscles for adult viability suggests a function of Gemin3 at the post-synaptic side. Based on the tissue-specific phenotypes uncovered, such a function is critical for pupal metamorphic changes and flight muscles. However, another possible explanation is that an earlier and wider disruption of Gemin3 by mesodermal-related drivers is responsible for the lethality, while late and local disruption of Gemin3 by neuroectodermal-related drivers causes milder phenotypes. More studies on the expression details of Gemin3 in pre- and post-synaptic tissues would help to distinguish those views.

### Relationship between Gemin3 and SMN

Studies in vertebrate systems, in vitro and in vivo, have shown that Gemin3 directly binds to SMN [24]. A recent study in *Drosophila* culture cells [23] and this study in fly tissues confirm that the interaction between Gemin3 and SMN is conserved from fly to human.

This study raises the possibility of a functional interaction between Gemin3 and SMN. Loss of *gemin3* phenocopies the larval mobility phenotypes observed in *smn* mutants [32]. Strong Gemin3 disruption in mesoderm and muscles led to striking developmental defects during metamorphosis, similar to those reported on disruption of SMN in a similar expression pattern [33]. A less severe *gemin3* disruption in the developing musculature results in viable but flightless adult flies, which have flight muscle degeneration, similar to the phenotype in a hypomorphic *smn* mutant [34].

We observed that *gemin3* mutants exhibit an overgrown NMJ before puparation and overexpression of *gemin3* leads to a significant decrease in NMJ area and branches relative to muscle size. Interestingly, two studies describe a range of NMJ phenotypes for *smn* mutants [32],[35]. It is still not clear whether *smn* and *gemin3* mutants have similar morphologic defects at the NMJ as the parameters and the segments used for NMJ analysis vary in different studies. Comparison of *smn* and *gemin3* mutant NMJs with the same standard, as well as analysing the NMJ phenotype in *smn* and *gemin3* double mutants would help to address this question.

The motor defects unravelled on disruption of Gemin3 function in *Drosophila* are very intriguing in view of its association with SMN, and the possible link to SMA. More studies are necessary to clarify the roles of SMN-Gemin3 interaction in development, which may help us to understand the molecular mechanisms of the devastating neurodegenerative disorder SMA.

## Materials and Methods

### Fly Stocks and Genetics

The *y w* stock was used as the wild-type control. Transposon insertion alleles *gemin3^R^* (P{PZ}Dhh1^rL562^) and *gemin3^W^* (*PBac*{RB}e03688) were obtained from the Bloomington *Drosophila* Stock Centre (BDSC) at Indiana University and the Exelixis collection at Harvard Medical School, respectively. Complementation tests, transposon remobilisation and rescue studies were carried out according to standard genetic crossing schemes. The RNAi transgenic constructs *UAS-gemin3^dwejra^* (49505) and *UAS-gemin3^munxar^* (49506) were obtained from the Vienna *Drosophila* RNAi Center and their generation was described in Dietzl et al. [36]. GAL4 lines used in this study included *1032*-GAL4, *Act5C*-GAL4 (BDSC), *elav*-GAL4 (BDSC), *nrv2*-GAL4 (gift from Paul Salvaterra, City of Hope National Medical Center, Duarte, California, USA), *D42*-GAL4 (BDSC), *OK6*-GAL4 (gift from Cahir O'Kane, University of Cambridge, Cambridge, UK), *C179*-GAL4 (BDSC), *how*-GAL4 (BDSC), *mef2*-GAL4 (gift from Barry Dickson, Research Institute of Molecular Pathology, Vienna, Austria), *G7*-GAL4 (gift from Aaron DiAntonio, Washington University, St. Louis, Missouri, USA) and *C57*-GAL4 (gift from Vivian Budnik, University of Massachusetts, Worcester, Massachusetts, USA); the spatial and temporal expression patterns are described in the Results. All stocks were cultured on standard molasses/maizemeal and agar medium in plastic vials or bottles at 25°C.

### *UAS-CFP::gemin3*, *UAS-gemin3*, and *UAS-gemin3^ΔN^* Transgenic Construct Generation

For the generation of the *P{CFP::gemin3}* transgenic construct, the PCR-amplified full-length coding sequence of *gemin3* was ligated into the *Kpn*I and *Xba*I restriction sites of the *pUAST* vector. The *Not*I and *Kpn*I restriction sites of the resulting recombinant vector were then used to insert the cyan fluorescent protein (CFP) coding portion of the pECFP-C1 vector (BD Biosciences Clontech, Palo Alto, California, USA) upstream of the *gemin3* sequence. The *P{UAS-gemin3}* construct was produced by ligating the *gemin3* cDNA (*Drosophila* Genomics Resource Centre, Indiana University) in the pUAST vector using the *Kpn*I and *Not*I restriction sites. The generation of the *P{UAS-gemin3^ΔN^}* involved PCR-amplification of the C-terminus of *gemin3* followed by ligation into the *Kpn*I and *Xba*I restriction sites of the *pUAST* vector. In both cases, the ligation products were used to transform *E. coli* competent cells using standard protocols. Correct transformants were further propagated and their harbouring plasmids were purified (Qiagen HiSpeed Plasmid Midi Kit, Qiagen Ltd., West Sussex, UK) prior to microinjection in *y w* embryos (BestGene Inc., Chino Hills, California, USA).

### RT-PCR

RNA was first extracted using the RNeasy kit (Qiagen Ltd.) and then reverse transcribed into cDNA using the QuantiTect Reverse Transcription Kit (Qiagen Ltd.) following manufacturer's instructions. PCR amplification of mRNA transcripts was performed using primers specific to *gemin3* (forward: 5′-CACTGGCCAAAATGGATCTAA-3′ and reverse: 5′-GGCATTGCCTCAATGAGTTT-3′) and *Tbp-1* (forward: 5′-CACCGAAAAGATCAAGGTCAA-3′ and reverse: 5′-CTTTGTTGACTCCGACCAGA-3′) mRNAs. RT-PCR products were resolved by electrophoresis on a 1.7% agarose gel containing ethidium bromide and bands were visualized by ultraviolet light.

### Behavioural Assays

Measurement of larval mobility involved placing age-matched larvae individually at the centre of a 0.7% agar plate and measuring the forward body wall contractions exhibited by each larva for 1 minute. Puparial axial ratios were calculated by dividing the length by the width of the puparia, both of which were measured from still images.

Adult viability assays were conducted by crossing GAL4 driver stocks to lines harbouring knockdown or truncated *gemin3* transgenes. A week following eclosion, adult flies were screened and counted. Adult viability was calculated as the percentage of the number of adult flies with the appropriate genotype divided by the expected number for the cross.

The flight assay was done according to a modified protocol originally designed by Benzer [37]. In brief, a 1000 ml-graduated cylinder divided into 5 sectors was coated internally with mineral oil. Flies were introduced into the top of the cylinder through a funnel and the flies stuck in each sector were counted. The height flies stick in the cylinder is indicative of their flight capabilities.

### Co-Immunoprecipitation and Western Blotting

Protein A beads washed and suspended in protein lysis buffer (2× protein lysis buffer [50 mM Tris pH8, 150 mM NaCl, 1 mM EDTA, and 1% v/v NP-40]+21× protease inhibitor cocktail [complete, Mini; Roche Diagnostics Ltd.]) were incubated with preimmune serum or an antigen-specific antibody, including rabbit anti-GFP (Abcam plc., Cambridge, UK) and rabbit anti-SMN (gift from Marcel van den Heuvel, University of Oxford). Sample lysates were prepared by dissecting body wall larval muscle fillets (∼30/IP) into cold 1× PBS followed by grinding into cold 2× protein lysis buffer. Following pre-clearing, lysates were incubated with beads coated with the appropriate target antigen-specific antibody. The beads were then washed in lysis buffer, and mixed with 4× NuPAGE LDS Sample Buffer (Invitrogen Ltd., Paisley, UK), 10× NuPAGE Reducing Agent (Invitrogen Ltd.) and deionised water. The mixture was then heated at 70°C in order to dissociate the immunoprecipitated antigen and any other macromolecules bound to it, followed by a brief spin. The bead-free supernatant was loaded onto a 4–12% NuPAGE Novex Bis-Tris pre-cast gel (Invitrogen Ltd.), resolved and probed for GFP according to standard Western blotting procedures.

### Immunostaining and Analysis of NMJs

Larvae were dissected in 1× PBS, fixed in 4% paraformaldehyde in PBS and then washed in 1× PBS+0.1% (v/v) Triton X-100 (PBT). The tissues were next subjected to overnight staining at 4°C by mouse anti-Discs large antibodies (1∶100; Developmental Studies Hybridoma Bank, University of Iowa, Iowa, USA). The next day, tissues were washed in PBT and stained for ∼2 hours at room temperature with anti-rabbit Alexa Fluor 488-conjugated secondary goat antibodies (1∶50), and anti-HRP goat antibodies conjugated to TRITC (1∶50; Jackson ImmunoResearch Laboratories Inc, West Grove, Pennsylvania, USA). Samples were then counterstained with nuclear-staining Hoechst 33342 (1∶500) and Cy5-conjugated actin-binding phallodin (1∶200) and mounted in Vectashield medium (Vector Laboratories Ltd., Peterborough, UK) prior to viewing with a Zeiss LSM 510 META confocal microscope.

ImageJ software (NIH) was used to quantify branch number, NMJ area, and muscle area from z-projections of confocal image stacks capturing ventral longitudinal muscles 6 and 7 (Segment A1). NMJ area constituted the presynaptic region stained by the anti-HRP antibody whereas branch number calculates the number of arborisations containing at least two boutons within a single NMJ. Both NMJ area and branch numbers were normalised through dividing each by the total muscle area of ventral longitudinal muscles 6 and 7.

### Histology of Adult Flight Muscles

Adult flies were fixed overnight in 4% (v/v) paraformaldehyde+2.5% (v/v) glutaraldehyde+0.1 M phosphate buffer pH7.2. The flies were then washed in 0.1 M phosphate buffer pH7.2 and post-fixed with 2% (w/v) osmium tetroxide for 2 hours at room temperature. Following a wash in water, the samples were subjected to a series of progressive dehydration steps in ethanol : water mixtures prior to embedding in Spurr's resin. Ultrathin sections were then made with a diamond knife, stained with Toluidine Blue and viewed under a light microscope.

## Supporting Information

Figure S1 The *gemin3* alleles. Schematic showing location and characteristics of the *gemin3* alleles. Sequence upstream of the *gemin3* transcription start site (black) flanks the *PBac*{RB} insert (red) of the *gemin3^W^* allele, whilst the sequence encoding the *gemin3* exon 1 fringes the *P*{PZ} insert (red) of the *gmn3^R^* allele. Transcribed but untranslated sequences are coloured in purple and the predicted translation of the *gemin3^R^* allele is also shown, with asterisks representing premature stop codons. On transposition, *P*-elements integrate into an 8 bp target site (underlined) that becomes duplicated at either end of the insertion. A schematic of the structure of each transposon construct is also shown [adopted from 54],[55]. The *P*{PZ} construct of the *gemin3^R^* allele has a plasmid backbone with an *E. coli* origin of replication (ori) and an antibiotic resistance gene (*kan*, *kanamycin*). The *PBac*{RB} construct of the *gemin3^W^* allele lacks some of the *piggyBac* 5′ and 3′ end sequences (denoted by square brackets). Both insert constructs are inserted in the reverse orientation.
(1.3 MB TIF)

Video S1 Loss of *gemin3* disrupts normal locomotive behaviour in third instar larvae. Compared to wild-type, *gemin3* heteroallelic mutants are sluggish in their movement.
(4.2 MB MOV)

Video S2 Gemin3 disruption in mesoderm and muscles results in loss of flight. Wild-type flies exhibit normal flight behaviour once tapped out of a plastic vial. In contrast, flies with an *mef2*-GAL3 driven expression of *gemin3^ΔN^* are flightless.
(1.8 MB MOV)
