# Supplementary figures and images for: A Motor Function for the DEAD-Box RNA Helicase, Gemin3, in Drosophila
Source: PLoS Genet. 2008 Nov 21;4(11):e1000265. doi: 10.1371/journal.pgen.1000265 (PMC2577925; doi:10.1371/journal.pgen.1000265)

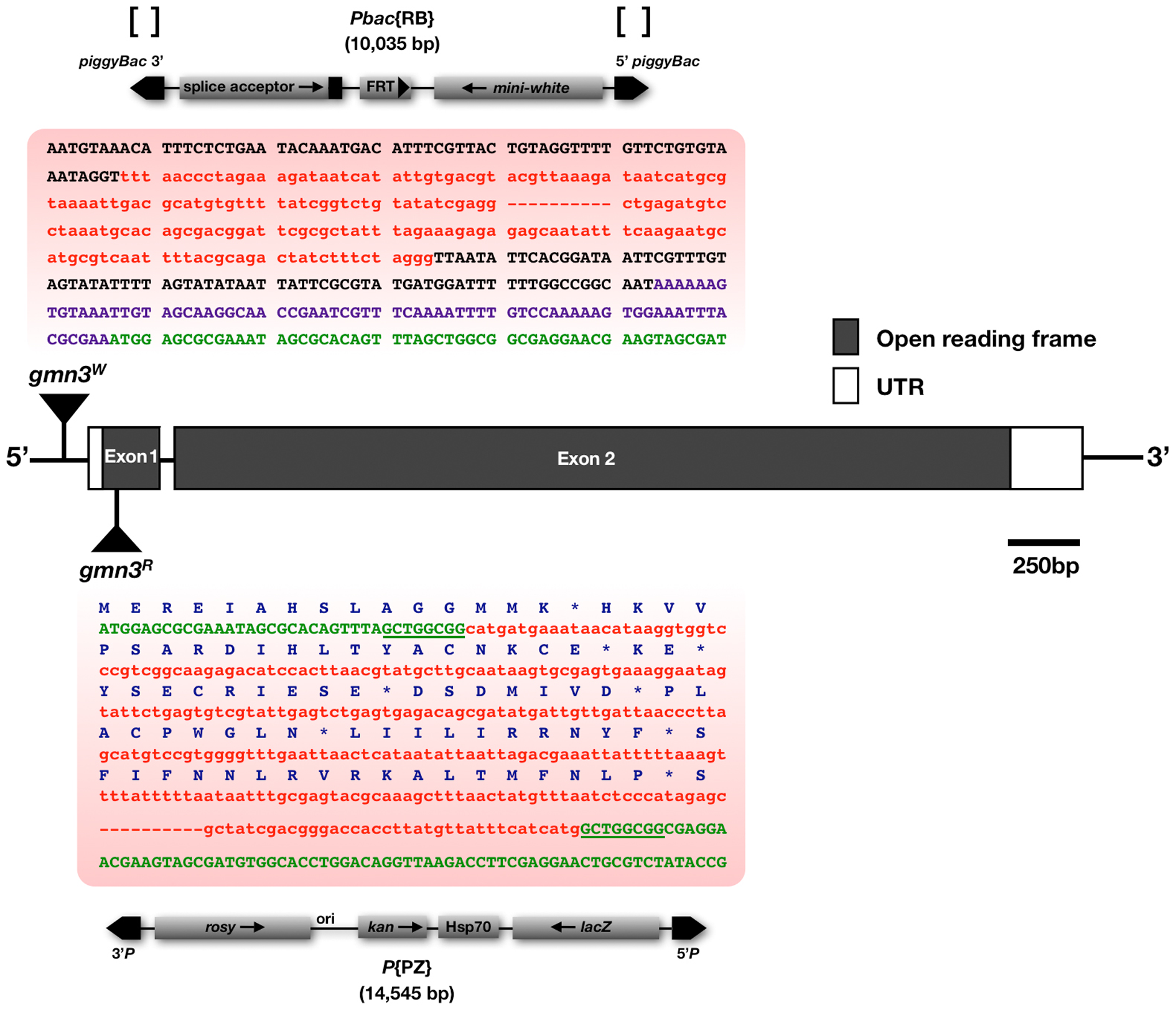

Supplement: Figure S1 — The gemin3 alleles. Schematic showing location and characteristics of the gemin3 alleles. Sequence upstream of the gemin3 transcription start site (black) flanks the PBac{RB} insert (red) of the gemin3W allele, whilst the sequence encoding the gemin3 exon 1 fringes the P{PZ} insert (red) of the gmn3R allele. Transcribed but untranslated sequences are coloured in purple and the predicted translation of the gemin3R allele is also shown, with asterisks representing premature stop codons. On transposition, P-elements integrate into an 8 bp target site (underlined) that becomes duplicated at either end of the insertion. A schematic of the structure of each transposon construct is also shown [adopted from 54],[55]. The P{PZ} construct of the gemin3R allele has a plasmid backbone with an E. coli origin of replication (ori) and an antibiotic resistance gene (kan, kanamycin). The PBac{RB} construct of the gemin3W allele lacks some of the piggyBac 5′ and 3′ end sequences (denoted by square brackets). Both insert constructs are inserted in the reverse orientation. (1.3 MB TIF) [file pgen.1000265.s001.tif]
